# Machine Learning for diagnosis of malignant thyroid nodules based on thyroid ultrasound: Systematic review and meta-analysis of studies with external datasets

**DOI:** 10.1016/j.ejro.2025.100716

**Published:** 2025-12-10

**Authors:** Elisa Gatta, Roberto Gatta, Riccardo Morandi, Samuele Isoli, Sara Corvaglia, Simone Vetrugno, Virginia Maltese, Ilenia Pirola, Claudio Casella, Carlo Cappelli

**Affiliations:** a Department of Clinical and Experimental Sciences, SSD Endocrinologia, University of Brescia, ASST Spedali Civili, Brescia, Italy; b Centro per la Diagnosi e Cura delle Neoplasie Endocrine e delle Malattie della Tiroide, University of Brescia, Brescia, Italy; c Department of Clinical and Experimental Sciences, University of Brescia, Brescia, Italy; d Department of Clinical and Experimental Sciences, Surgical Clinic, University of Brescia, ASST Spedali Civili, Brescia, Italy; e Department of Internal Medicine and Therapeutics, University of Pavia, Pavia, Italy

**Keywords:** Thyroid nodules, Machine learning, Artificial intelligence

## Abstract

**Introduction:**

Optimizing the diagnostic approach to thyroid nodules remains a crucial challenge. Ultrasound-based risk stratification systems such as EU-TIRADS have shown reasonable sensitivity and specificity. Therefore, we conducted a systematic review and meta-analysis to assess the diagnostic performance of Artificial Intelligence (AI) models in differentiating benign from malignant thyroid nodules on ultrasound data.

**Methods:**

A comprehensive search of PubMed/MEDLINE, Scopus, and Web of Science was performed up to January 1, 2025. Eligible studies included patients with thyroid nodules undergoing ultrasound, where AI-based models were validated against cytological or histological findings. The AI algorithms were developed using different types of ultrasound-derived data, including B-mode images, radiomics features. Pooled sensitivity, specificity, and area under the curve (AUC) were estimated using a hierarchical summary receiver operating characteristic (HSROC) model.

**Results:**

Twenty-seven studies comprising 146,332 patients and over 600,000 ultrasound images met inclusion criteria. Overall, pooled sensitivity was 87 % (95 % CI: 84–89 %) and specificity 83 % (95 % CI: 79–86 %). The summary operating point indicated a sensitivity of 88 % and specificity of 83 %, with an AUC of 91.9 % (95 % CI: 90.0–93.2 %). Although subgroup analysis suggested higher accuracy when cytology was used as the reference standard compared to histology, the mixed-effects meta-regression did not confirm a statistically significant association (p = 0.238 for sensitivity; p = 0.188 for specificity).

**Conclusion:**

AI-based algorithms show excellent diagnostic performance in distinguishing benign from malignant thyroid nodules, with robust validation across external datasets. These findings support the potential integration of AI into clinical thyroid nodule management, although further multicenter, non-Asian, and histology-based studies are warrantee.

**Systematic review registration:**

PROSPERO (CRD420251108149)

## Highlights

• AI shows high accuracy in thyroid nodule malignancy detection.
• Pooled sensitivity 87 % and specificity 83 % across 27 studies.
• Cytology- and histology-based validations yielded comparable diagnostic performance.
• Results support AI integration into thyroid nodule management.

## 1 Introduction

Ultrasound is the cornerstone imaging modality for the evaluation of thyroid owing to its wide availability, safety, and real-time capability [1], [2]. Conventional B-mode imaging provides high-resolution morphological information, allowing assessment of echogenicity, margins, and internal architecture. Doppler imaging complements this by evaluating vascular patterns, which may reflect tissue perfusion and inflammatory or neoplastic activity [3]. More recently, elastography techniques—both strain and shear-wave—have enabled quantitative assessment of tissue stiffness, offering additional diagnostic insights [4], [5], [6]. Thyroid nodules (TNs) are highly prevalent in the general population, particularly among women and older adults [7]. Given their frequency and the potential for underlying malignancy, international guidelines uniformly advocate prompt ultrasound risk stratification at diagnosis [8]. Despite these strengths, ultrasound remains inherently operator-dependent and subject to artifacts that may limit reproducibility and inter-observer agreement [9].

Moreover, conventional ultrasound-based risk stratification systems, although standardized, still rely on subjective visual interpretation and may yield inconsistent performance across centers and operators. The inter-observer variability in describing sonographic features such as echogenicity or margins remains a key limitation of the current approach to thyroid nodule management [9].

The optimization of diagnostic and therapeutic strategies has long been a central objective in medical practice. In the field of thyroidology, particularly in the selection of nodules for cytological evaluation, several ultrasound-based scoring systems have been developed to identify lesions suitable for fine-needle aspiration, demonstrating satisfactory sensitivity and specificity [8], [10], [11]. Among these, a recent meta-analysis by Yang et al. encompassing 88 studies and 59,304 nodules reported a sensitivity of 75 % and a specificity of 82 % for EU-TIRADS category TR5. Notably, specificity decreased substantially for categories TR4 and TR3, reaching 62 % and 31 %, respectively [12].

The concept of using computers to simulate intelligent behavior, later termed AI, was first introduced by Alan Turing in the 1950s [13]. Since then, this field has undergone a dramatic evolution, with profound implications for medicine. In particular, the application of AI to medical imaging has been proposed as a strategy to enhance diagnostic accuracy, consistency, and efficiency. In 2017, the U.S. Food and Drug Administration approved the first cloud-based deep learning application for clinical use in healthcare [14], marking a turning point in the integration of AI into routine practice. Deep learning has since been applied to lesion detection, differential diagnosis generation, and automated reporting, impacting almost all areas of medicine [14]. In thyroidology, one of the earliest applications of AI to ultrasound imaging was reported in the 1990s, when Karakitsos et al. employed a back-propagation neural network to assist clinicians in distinguishing between benign and malignant thyroid nodules [15].

AI is increasingly applied in medicine, offering promising opportunities for improving diagnostic accuracy and workflow efficiency, although its clinical adoption remains limited. Its applications extend well beyond professional domains, influencing everyday life and clinical practice alike. AI was first applied to thyroidology in the 1990s, and its potential is now being increasingly investigated across a growing body of studies [16], [17].

Radiomics, a promising branch of AI, enables the automated extraction of large volumes of quantitative data from medical images. In the context of thyroid nodules, radiomics has demonstrated the ability to detect imaging features imperceptible to the human eye, thereby improving risk stratification, supporting differentiation between benign and malignant lesions, and informing decision-making regarding fine-needle aspiration. By integrating imaging-derived data with advanced algorithms and predictive models, radiomics may promote a more accurate, personalized, and non-invasive approach to thyroid nodule management [18], [19], [20]. However, current AI-based models also present relevant challenges, including the need for large annotated datasets, lack of model interpretability (“black-box” nature), and limited generalizability across populations and imaging devices. Conversely, their main strength lies in the ability to quantify subtle imaging features beyond human perception, potentially supporting more objective and reproducible risk stratification (BIBLIO).

The aim of this systematic review and meta-analysis was to quantitatively assess the diagnostic accuracy of AI models—validated on external, independent datasets—in differentiating benign from malignant thyroid nodules, using cytological or histological diagnosis as the reference standard.

## 2 Material and methods

### 2.1 Search strategy and inclusion criteria

A wide literature search of the PubMed/MEDLINE, Scopus and Web of Science databases was made.

The review questions were defined according to the diagnostic accuracy framework (Population, Index test, Reference standard, and Diagnosis):

− Population: patients with thyroid nodules who underwent ultrasound examination;
− Index test: artificial intelligence–based models (including machine learning and radiomics) applied to ultrasound data;
− Reference standard: cytological and/or histological findings;
− Diagnosis (target condition): discrimination between benign and malignant thyroid nodules.

The algorithm used for the research was the following: (“artificial intelligence” OR “machine learning” OR “deep learning” OR “neural network” OR “decision tree” OR “random forest” OR “nearest neighbor” OR “naive Bayes” OR “support vector machine”) AND (“diagnosis” OR “screen*” OR “classifi*” OR “discriminat*” OR “performance” OR “sensitivity” OR “specificity” OR “accuracy” OR “area under the curve” OR “AUC” OR “calibrat*”) AND (“thyroid” OR “thyroid gland”) AND (“cancer” OR “neoplasm*” OR “carcinoma” OR “nodule*” OR “tumor*” OR “tumour*” OR “malignan*” OR “adenoma”).

The search was updated until January 1, 2025. Only articles in English were considered, and preclinical studies, conference proceedings, reviews, or editorials were excluded. To expand our search, the references of the retrieved articles were also screened for additional papers; moreover, other studies were identified by looking through all the articles that cite the papers included in the review ("snowballing"). All eligible studies were exported and managed in EndNote 20.3.

### 2.2 Eligibility criteria

The eligibility criteria were chosen taking into account the review question. Studies addressing the review questions defined according to the PICO framework were included. Exclusion criteria for the systematic review (qualitative analysis) were reviews, letters, comments, or editorials on the topic of interest, on the analyzed topic (as these articles are characterized by poor-quality evidence and are typically affected by publication bias), and original studies unrelated to the review question, such as those evaluating AI models applied to cytology, pathology, or non-ultrasound imaging data.

Studies were included if they assessed the diagnostic performance of artificial intelligence models using ultrasound-based inputs with cytological or histological confirmation as the reference standard. All indeterminate or equivocal diagnoses, whether histological or cytological (e.g., Bethesda III and IV), were excluded from the analysis. In addition, studies in which the diagnostic reference standard consisted solely of an ultrasound-based risk score (i.e., TI-RADS) without cytological and/or histological confirmation were excluded.

To ensure methodological homogeneity, studies in which clinical data were integrated as a major component of the model were excluded.

### 2.3 Study selection

E.G. and C.C. independently read the titles and abstracts of the records generated by the search algorithm. They then determined which studies were eligible based on predefined criteria. Discrepancies were resolved through discussion, and a third reviewer (R.G.) was consulted in case of disagreement.

E.G. is currently pursuing a Ph.D. in Artificial Intelligence and obtained her medical degree five years ago; she has also recently completed her specialization in Endocrinology. C.C. is a board-certified endocrinologist with twenty years of specialty experience and currently serves as Professor of Endocrinology. R.G. holds a Ph.D. in Oncological Science and serves as a researcher, with primary research interests in radiomics and process mining for healthcare.

### 2.4 Reporting

**Fig. 1:**
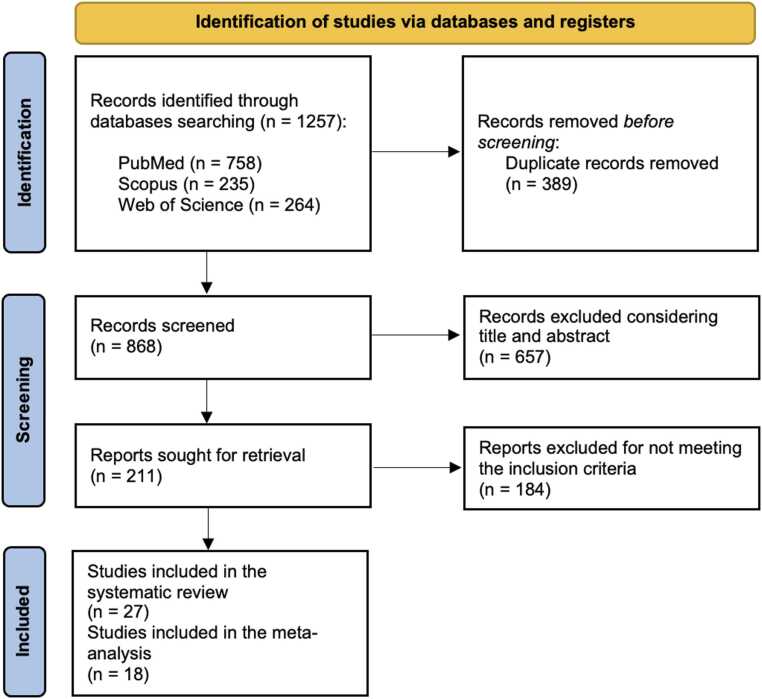
Flowchart of the study selection process for eligible studies.

**Fig. 2:**
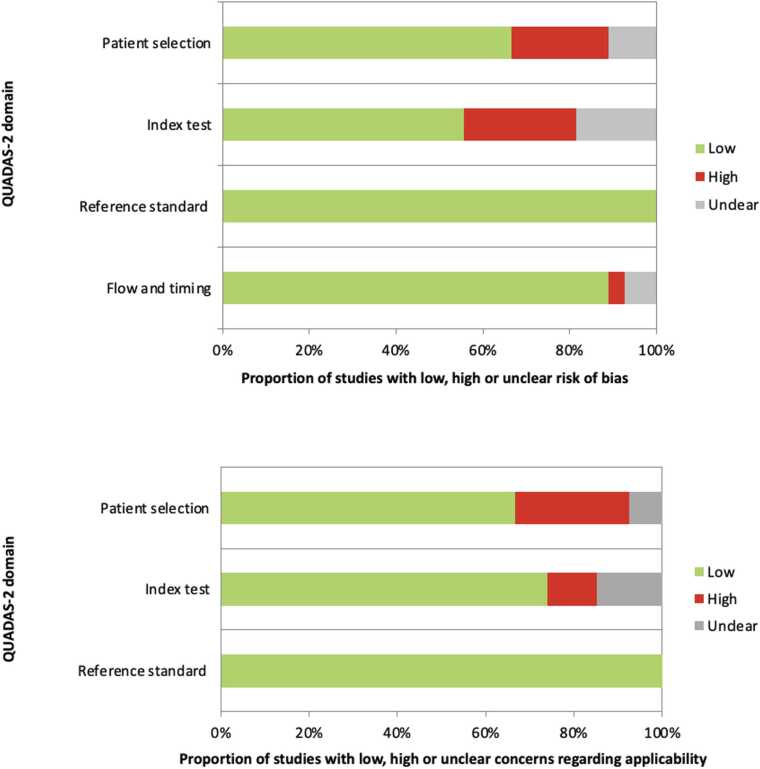
QUADAS-2 assessment of risk of bias and applicability concerns for the studies included in the review.

**Fig. 3:**
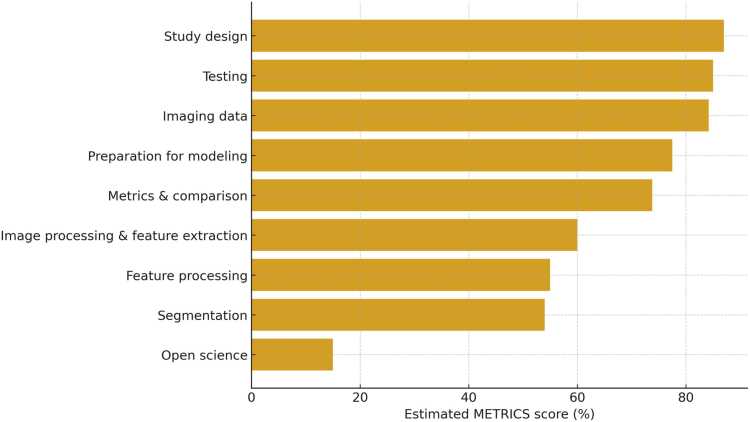
Distribution of METhodological RadiomICs Score (METRICS) domain scores across the included studies.

The protocol of this systematic review is registered in PROSPERO (CRD420251108149) and followed the PRISMA (Preferred Reporting Items for Systematic Reviews and Meta-Analyses) guidelines [21] (Fig. 1). The quality assessment of the studies, including the risk of bias and applicability concerns, was carried out using Quality Assessment of Diagnostic Accuracy Studies version 2 (QUADAS-2) evaluation [22] (Fig. 2). The methodological quality of the included studies was evaluated using the METhodological RadiomICs Score (METRICS) [23] (Fig. 3).

### 2.5 Data extraction

The reviewers collected data from all included studies by examining the full text, tables, and supplementary materials. Extracted data covered general study information (authors, publication year, country, study design, clinical setting), patient characteristics (sample size, inclusion/exclusion criteria, relevant clinical features), reference standard (histological and/or cytological confirmation), type of internal validation, number of images, and automated nodule localization. The main findings of the articles included in this review are reported in the Results section

### 2.6 Statistical analysis

To assess the diagnostic performance of the machine learning algorithm, pooled meta-analyses of sensitivity and specificity were conducted using a bivariate random-effects model. In addition, pooled diagnostic odds ratio (DOR), positive likelihood ratio (DLR+), and negative likelihood ratio (DLR–) were calculated. A mixed-effects meta-regression model was applied to explore potential moderators accounting for between-study heterogeneity, specifically to assess differences in diagnostic performance between studies adopting cytological versus histological outcomes as the reference standard. Publication bias was assessed using Deeks’ funnel plot asymmetry and the associated weighted regression test. A hierarchical summary receiver operating characteristic (HSROC) curve was generated, including pooled sensitivity, specificity, area under the curve (AUC) values with corresponding 95 % confidence intervals (CIs), summary operating point and prediction regions. All analyses were performed using RStudio (version 2025.05.1 +513), with a significance level set at p < 0.05.

## 3 Results

### 3.1 Literature search

A total of 1257 articles were identified through the computerized literature search. After removal of duplicates, 868 articles remained. By screening titles and abstracts, 657 articles were excluded as their content was not relevant to the focus of this review. “Consequently, 211 articles were retrieved for full-text evaluation, of which 184 were excluded because they did not meet the inclusion criteria of our study. In particular, most of them lacked an external dataset for algorithm validation (Fig. 1). Therefore, 27 studies were ultimately included in the review [24], [25], [26], [27], [28], [29], [30], [31], [32], [33], [34], [35], [36], [37], [38], [39], [40], [41], [42], [43], [44], [45], [46], [47], [48], [49], [50]. Among these, only 18 studies provided complete diagnostic data (true positives, false negatives, false positives, and true negatives), allowing the construction of 2 × 2 contingency tables and their inclusion in the quantitative meta-analysis.

In general, the quality assessment using QUADAS-2 evaluation underlined the presence of unclear risk of bias and applicability concerns in some of the studies for what concerns patients’ selection, index test, reference standard and flow and timing (Fig. 2). Several studies did not clearly report whether patients were enrolled consecutively or randomly; in addition, exclusion criteria were often poorly described, contributing to the high risk of bias in this domain. Moreover, several papers did not specify whether the threshold for malignancy was pre-specified or derived post-hoc, which represents a methodological concern in diagnostic accuracy studies. The quality assessment using the METRICS revealed moderate overall methodological rigor among the included studies, with the highest scores observed in the domains of study design and testing, and lower adherence in the areas of segmentation, feature processing, and open-science practices (Fig. 3).

### 3.2 Characteristics of the studies

The main characteristics and results of the included studies are summarized in Table 1. In total, 146,332 patients were evaluated. Of the 27 studies, 24 (133,813 patients) were retrospective, 2 (11,755 patients) were retrospective–prospective and 1 prospective (764 patients). Eighteen studies compared machine learning performance against histopathological findings, while nine used cytological outcomes from fine-needle aspiration as the reference standard.

**Table 1:** Characteristics of the human studies considered for the review.

**First author**	**Ref. N.**	**Year**	**Study design**	**N. of patients**^**a**^	**N. of images**^**a**^	**Reference standard (histological and/or cytological) for external testing set**	**AI Method used**	**Type of internal validation**	**Automated nodule localization**
Li	[24]	2019	Retrospective study	44,070/154/1420	312,399/8606/NR/741; 11,039	Histological	DL – CNN	NA	Yes
Song	[25]	2019	Retrospective study	NA	1358/NR/55/100	Cytological	DL – TL	Holdout cross-validation	No
Song	[26]	2019	Retrospective study	1580/299	6228/NR/367/152	Histological	DL – CNN	Five-fold cross-validation	Yes
Bai	[27]	2020	Retrospective study	NA	14,531/NR/3633/437; 570	Histological	DL – RS-Net; CNN	Five-fold cross-validation	No
Koh	[28]	2020	Retrospective study	14,194/781/200/200	13,560/NR/634/781; 200; 200	Histological	DL – CNN	NA	No
Wei	[29]	2020	Retrospective study	11,604/261	17,859/NR/7650/1032	Histological	DL	Random split-sample validation	Yes
Zhou	[30]	2020	Retrospective study	1629/105	1097/547/NR/105	Cytological	DL – CNN; TL	NA	Yes
Peng	[31]	2021	Retrospective–prospective study	8339/1428/1048/303	14,439/3610/NR/4305	Histological	DL – RS-Net	NA	Yes
Wu	[32]	2021	Retrospective study	1396/197	1146/NR/143/112 698/NR/95/101 1844/NR/238/213	Histological	DL – CNN	Random split-sample validation	No
Zhu	[33]	2021	Retrospective study	6426/261	16,401/1000/300/1032	Histological	DL – CNN	NA	Yes
Chen	[34]	2022	Retrospective study	450/186	1076/269/NR/243	Histological	DL – CNN	Five-fold cross-validation	No
Deng	[35]	2022	Retrospective study	NA	3125/391/391/831	Histological and cytological	DL – CNN; MTL	Random split-sample validation	Yes
Han	[36]	2022	Retrospective study	3096/886	2344/781/781/886	Cytological	MTL DenseNet	Ten-fold cross-validation	No
Keutgen	[37]	2022	Retrospective study	NA	734/184/68/66	Histological and cytological	ML	Five-fold cross-validation	No
Kim	[38]	2022	Retrospective study	7518/59	12,327/3082/432/168	Histological	DL – CNN	Random split-sample validation	Yes
Zhang	[39]	2022	Retrospective study	NA/400/587	NR	Histological	DL – CNN	Ten-fold stratified cross-validation	No
Chen	[40]	2023	Retrospective study	485/80	1012/253/NR/126	Histological and cytological	DL – CNN	Three-fold cross-validation	No
Gao	[41]	2023	Retrospective study	NA/NA	4989/NR/NR/309	Histological	MTL	Five-fold cross-validation	No
Tang	[42]	2023	Retrospective study	NA	7700/1923/NR/431	Histological	TS-DSANN	Five-fold cross-validation	No
Xu	[43]	2023	Retrospective study	10,023 (I+E)	18,477/4563/1904 (I+E)	Histological	AI	Random split-sample validation	No
Yang	[44]	2023	Retrospective study	432/71	1392/349/NR/309	Histological	DL – CNN	Random split-sample validation	No
Yao	[45]	2023	Retrospective study	1349/163/178	1349/NR/NR/163; 178	Histological	DL – CNN,	Ten-fold cross-validation	Yes
Yao	[46]	2023	Retrospective study	7460/619	8017/1002/NR/1002	Histological	DL – CNN	NA	Yes
Chen	[47]	2024	Retrospective study	6401/138	7636/1097/2129/339	Histological	DL – CNN	NA	Yes
Feng	[48]	2024	Retrospective study	7881/574	16,906/NR/1130/1262	Histological and cytological	DL	NA	No
Wu	[49]	2024	Prospective study	672/92	38,336/5376/10,368/8064	Histological and cytological	DL – CNN, ST	NA	Yes
Zhou	[50]	2024	Retrospective–prospective study	346/291	NR/NR/977/906	Histological and cytological	AI	NA	No

Ref.: references; N.: number; AI: artificial intelligence; NA: not available; DL: deep learning; CNN: Convolutional neural network; TL: transfer learning; RS-Net: risk stratification network; MTL: Multitask Learning; TS-DSANN: texture and shape focused dual-stream attention neural network; ST: swin-transformer

^a^ Internal test/external test/external test/external test

In all eligible studies, sonographic data were retrospectively extracted from institutional picture archiving and communication systems (PACS). The vast majority of AI models were trained on B-mode ultrasound images representing the largest longitudinal and/or transverse planes of each thyroid nodule. When applicable, the region of interest (ROI) was either manually or automatically segmented before feature extraction or network training. Approximately one third of the studies implemented radiomics pipelines to derive quantitative descriptors of texture, shape, and gray-level distribution from these ROIs.

Overall, 524,438 images were used for training, and 100,084 images for external validation. All studies were conducted in Asia.

When studies were stratified by imaging modality and AI framework, distinct performance patterns emerged, revealing how algorithm design and ultrasound input influence diagnostic reliability.

Models trained on static B-mode images using conventional convolutional neural networks (CNNs) achieved pooled AUCs ranging from 0.83 to 0.94, with sensitivity typically between 85–93 % and specificity between 82–94 % [29], [36]. These models demonstrated robust discrimination, especially in large multicenter datasets, yet their outputs remained largely black-box and prone to variability when applied across ultrasound systems or acquisition protocols.

By contrast, knowledge-guided architectures integrating TI-RADS features [24], [32] or interpretable frameworks [46] maintained comparable accuracy (AUC 0.90–0.93) but introduced an important conceptual advance: they linked each AI-derived probability to interpretable sonographic descriptors such as composition, echogenicity, or margins. This mechanistic transparency mitigates interobserver heterogeneity and enhances trust in AI-assisted decision support, representing a crucial step toward regulatory acceptance.

Three studies directly compared AI-assisted ultrasound with conventional sonographic interpretation by radiologists using TI-RADS or equivalent descriptors. In Chen et al., the AI model for thyroid nodule classification based on multitask deep learning using TI-RADS characteristics achieved an AUC of 0.91, sensitivity of 83 %, and specificity of 87 %, which was comparable to experienced radiologists (AUC 0.93; sensitivity 92 %) but with significantly higher specificity (80 %, p = 0.02), and clearly superior to junior readers (AUC 0.78; sensitivity 70 %; specificity 75 %) [34]. Similarly, in an effective and result-interpretable and-to-and thyroid nodule classification network, the AI system outperformed both intermediate and senior radiologists on internal and external validation datasets, with statistically significant differences in AUC according to DeLong’s test [36]. Another study employing an ensemble deep-learning classifier (EDLC-TN) reported AI accuracy and AUC equal to or higher than those of individual radiologists, with further improvement when AI outputs were integrated with human interpretation [29].

Studies using multimodal or video-based ultrasound further improved performance by incorporating dynamic and contextual cues—such as vascularity, tissue elasticity, and temporal consistency—achieving the highest pooled accuracy (AUC ≈ 0.97, sensitivity ≈ 0.90, specificity ≈ 0.94) [49]. These results indicate that temporal information, often neglected in static-image datasets, carries incremental diagnostic value by approximating real-time expert evaluation.

Finally, transformer-based models leveraging self-attention mechanisms showed stable generalization across centers, sexes, and ultrasound devices (AUC ≈ 0.94) [48]. Their performance consistency suggests that attention-based architectures may capture higher-order spatial dependencies less sensitive to vendor-related variation.

### 3.3 Quantitative analysis

**Fig. 4:**
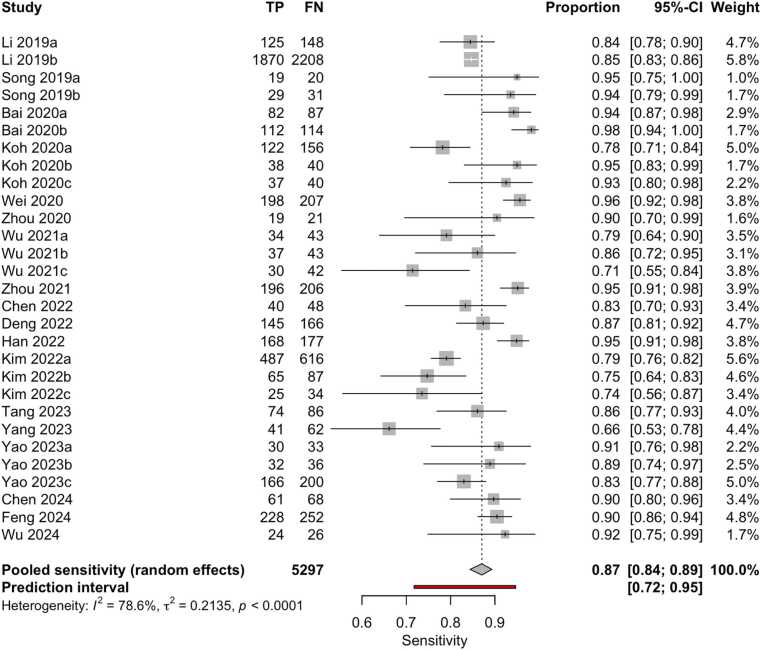
Forest plot of sensitivity estimates from studies evaluating machine learning algorithms for the diagnosis of thyroid carcinoma, using either histological or cytological findings as the reference standard and tested on external datasets.

**Fig. 5:**
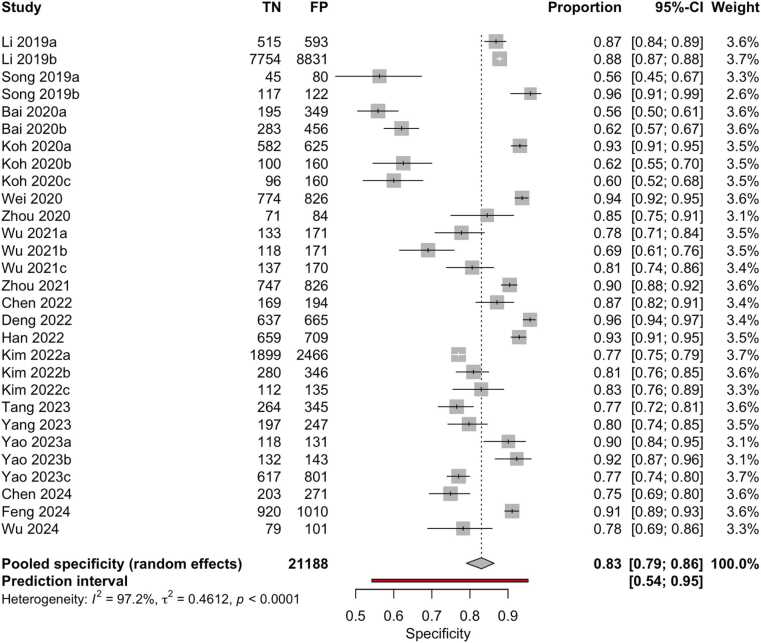
Forest plot of specificity estimates from studies evaluating machine learning algorithms for the diagnosis of thyroid carcinoma, using either histological or cytological findings as the reference standard and tested on external datasets.

Although 18 studies were included in the quantitative synthesis, the number of datapoints shown in the forest plots (Fig. 4, Fig. 5) is higher. This discrepancy arises because several studies assessed the same AI model on multiple independent external datasets, each yielding separate diagnostic performance metrics. As reported in Table 1 (column ‘I/E/E/E’), these datasets were extracted and analyzed individually to preserve their distinct outcomes. Accordingly, the total number of evaluations represented in the meta-analysis exceeds the number of included papers.

**Fig. 6:**
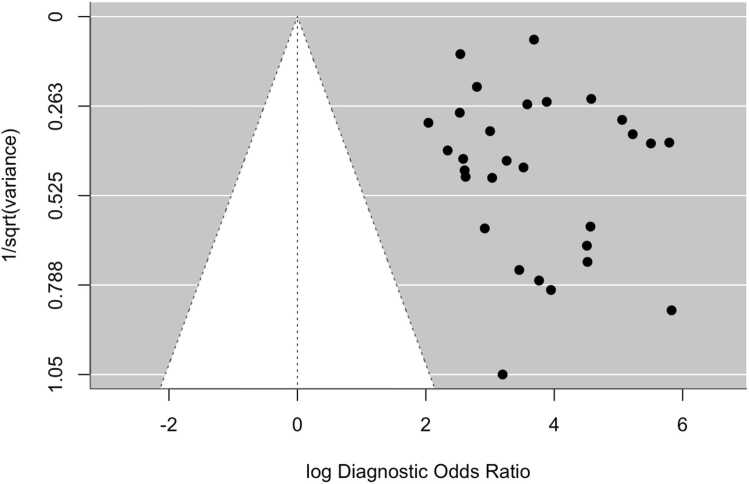
Deeks’ funnel plot for publication bias of studies using histological or cytological findings as the reference standard and tested on external datasets.

**Fig. 7:**
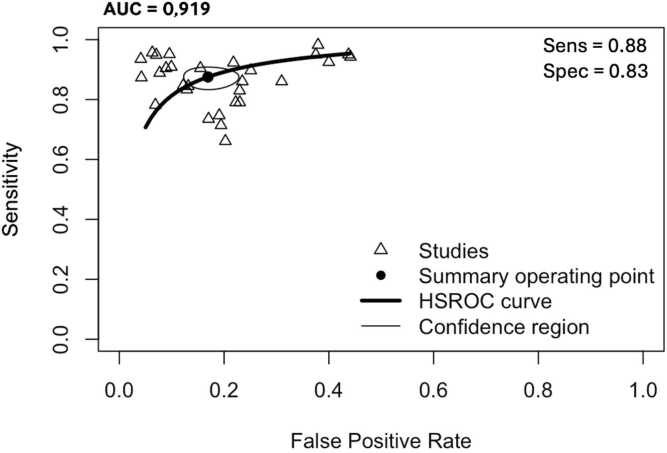
Hierarchical summary receiver operating characteristic curve of machine learning algorithms for the diagnosis of thyroid carcinoma, using histological or cytological findings as the reference standard and tested on external datasets.

Overall, the aggregated sensitivity of all included studies was 87 % (95 % CI: 84–89 %) (Fig. 4), while the specificity was 83 % (95 % CI: 79–86 %) (Fig. 5). Based on these pooled estimates, the DLR+ was 5.1, the DLR– was 0.16, and the DOR was 32.7. The Deeks’ funnel plot for publication bias (Fig. 6) shows a relatively symmetric distribution of studies around the regression line, suggesting the absence of major small-study effects. The regression test for funnel plot asymmetry confirmed this visual impression, yielding a non-significant result (t = 0.67; p = 0.51). The corresponding area under the curve (AUC) was 91.9 % (95 % CI: 90.0–93.2 %), with a summary operating point indicating a sensitivity of 88 % and a specificity of 83 % (Fig. 7).

To investigate potential bias related to the diagnostic reference standard (cytological vs. histological data), we performed a subgroup analysis.

**Fig. 8:**
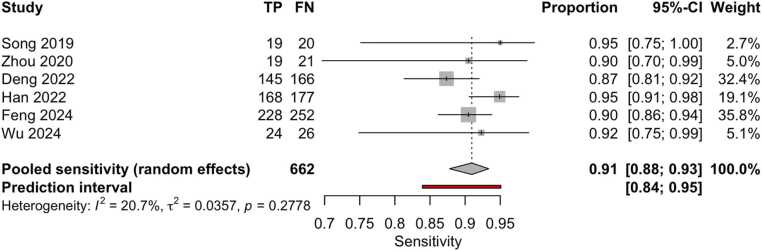
Forest plot of sensitivity estimates from studies evaluating machine learning algorithms for the diagnosis of thyroid carcinoma, using cytological findings as the reference standard and tested on external datasets.

**Fig. 9:**
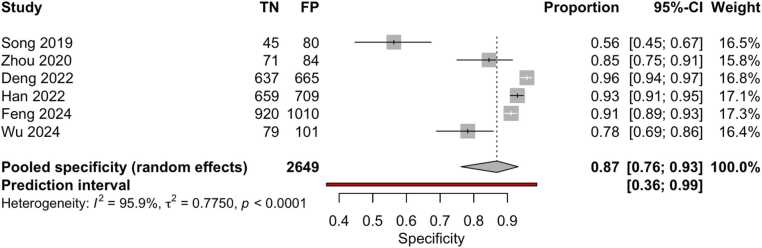
Forest plot of specificity estimates from studies evaluating machine learning algorithms for the diagnosis of thyroid carcinoma, using cytological findings as the reference standard and tested on external datasets.

**Fig. 10:**
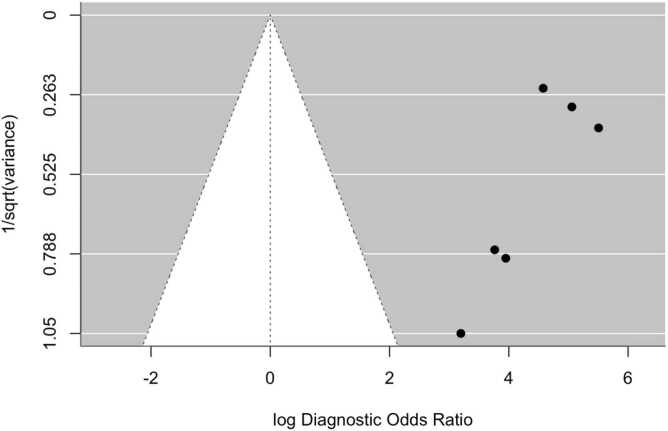
Deeks’ funnel plot for publication bias of studies using cytological findings as the reference standard and tested on external datasets.

**Fig. 11:**
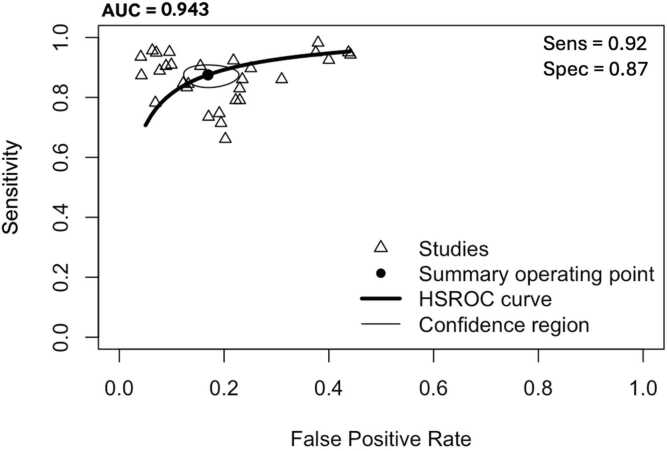
Hierarchical summary receiver operating characteristic curve of machine learning algorithms for the diagnosis of thyroid carcinoma, using cytological findings as the reference standard and tested on external datasets.

Six studies (15,572 patients, 95,100 images) compared machine learning results with cytological outcomes [25], [30], [35], [36], [40], [48], [49]. The pooled sensitivity was 91 % (95 % CI: 88–93 %) (Fig. 8), and specificity was 87 % (95 % CI: 76–93 %) (Fig. 9). Based on these pooled estimates, the DLR+ was 7.0, the negative likelihood ratio DLR–was 0.10, and the DOR was 70.0. The Deeks’ funnel plot (Fig. 10) illustrates a nearly symmetric distribution of studies around the regression line, without substantial evidence of small-study effects. The regression test for funnel plot asymmetry confirmed the absence of significant publication bias (t = –1.26; p = 0.28). The AUC was 94.3 % (95 % CI: 92.4–96.0 %), with a summary operating point corresponding to a sensitivity of 92 % and a specificity of 87 % (Fig. 11).

**Fig. 12:**
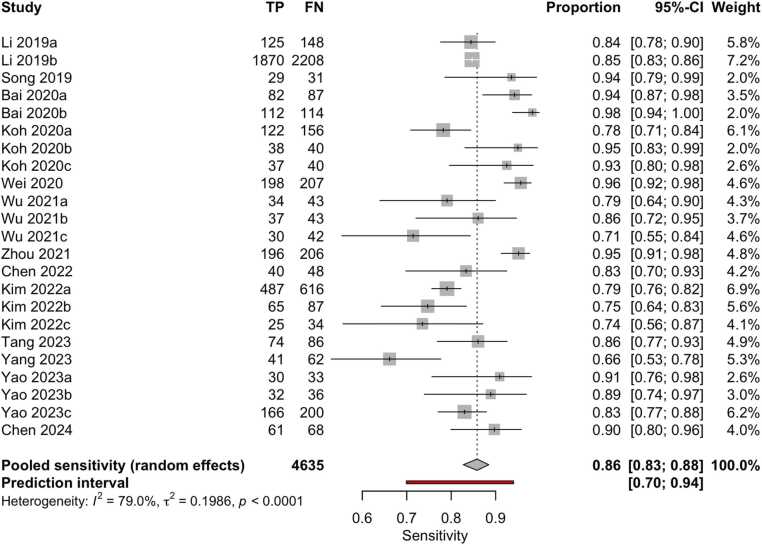
Forest plot of sensitivity estimates from studies evaluating machine learning algorithms for the diagnosis of thyroid carcinoma, using histological findings as the reference standard and tested on external datasets.

**Fig. 13:**
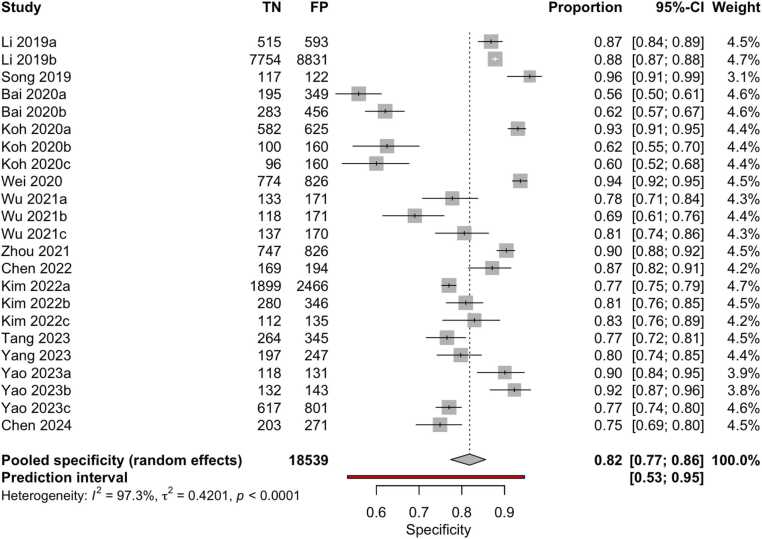
Forest plot of specificity estimates from studies evaluating machine learning algorithms for the diagnosis of thyroid carcinoma, using histological findings as the reference standard and tested on external datasets.

**Fig. 14:**
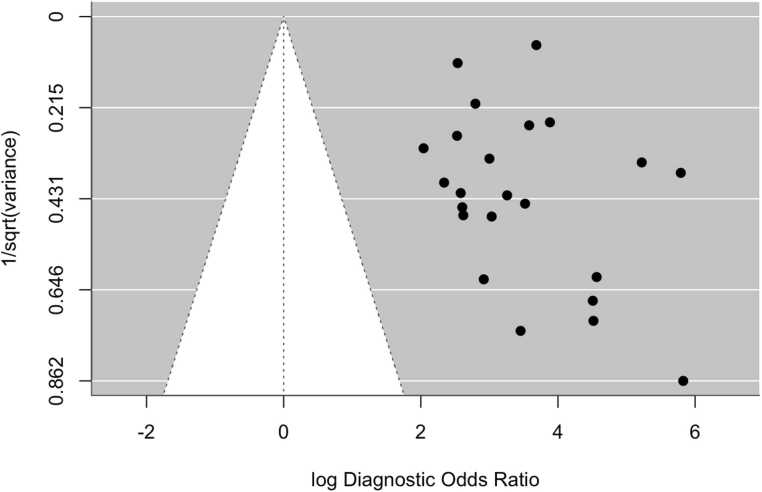
Deeks’ funnel plot for publication bias of studies using histological findings as the reference standard and tested on external datasets.

**Fig. 15:**
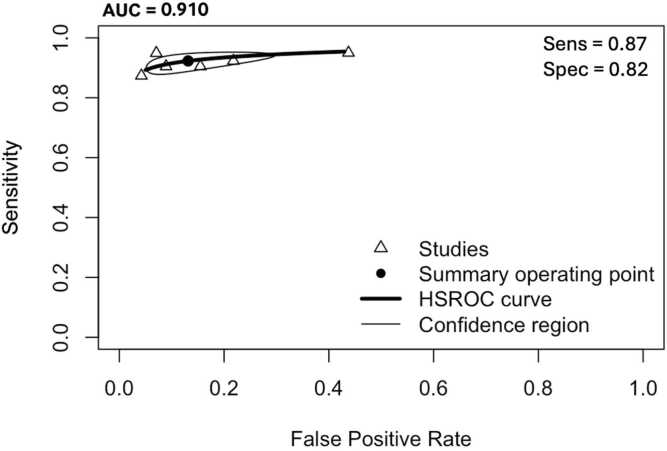
Hierarchical summary receiver operating characteristic curve of machine learning algorithms for the diagnosis of thyroid carcinoma, using histological findings as the reference standard and tested on external datasets.

Fourteen studies (108,067 patients, 474,383 images) compared results with histopathological findings [24], [26], [27], [28], [29], [32], [33], [34], [38], [42], [44], [45], [46], [47]. The pooled sensitivity was 86 % (95 % CI: 83–88 %) % (Fig. 12), and specificity was 82 % (95 % CI: 77–86 %) (Fig. 13). Based on these pooled estimates, the DLR+ was 4.8, the DLR– was 0.17, and the DOR was 28.2. The Deeks’ funnel plot (Fig. 14) shows a symmetric distribution of studies around the regression line, indicating the absence of relevant small-study effects or selective publication. The regression test for funnel plot asymmetry confirmed this visual impression, yielding a non-significant result (t = 0.07; p = 0.95). The AUC was 91.0 % (95 % CI: 89.7–93.2 %), with a summary operating point corresponding to a sensitivity of 87 % and a specificity of 82 % (Fig. 15).

Although the subgroup analysis suggested higher accuracy when cytology was used as the reference standard compared to histology, the mixed-effects meta-regression did not confirm a statistically significant association (p = 0.238 for sensitivity; p = 0.188 for specificity). The regression coefficient for logit sensitivity was β = –0.071 (95 % CI: –0.19–0.05) and for logit specificity β = –0.090 (95 % CI: –0.22–0.04). The residual heterogeneity was negligible for sensitivity (τ² = 0) and low for specificity (τ² = 0.013), indicating that the moderator variable did not meaningfully account for between-study variability.

### 3.4 Discussion

The present systematic review and meta-analysis highlights the excellent diagnostic performance of machine learning algorithms in differentiating benign from malignant thyroid nodules, with pooled sensitivities and specificities of 87 % and 83 %, respectively, validated in external datasets.

These findings align with the expanding literature supporting the application of AI in thyroid ultrasound imaging [51], [52], [53], [54]. Jassal et al. recently highlighted the burgeoning potential of AI in the clinical management of cytologically indeterminate thyroid nodules; however, they emphasized that most available studies lacked robust, independent external validation [55]. In contrast, the present study, which focused on AI applications in differentiating benign from malignant thyroid nodules, demonstrated consistently good accuracy across external datasets.

To date, ultrasound-based risk stratification systems relying on nodular features have been established to identify lesions suspected of malignancy and to improve interobserver agreement. A large meta-analysis by Yang et al., including 88 studies and 59,304 nodules, reported a sensitivity of 75 % and a specificity of 82 % for EU-TIRADS category TR5. Notably, specificity declined substantially for TR4 and TR3 categories, reaching 62 % and 31 %, respectively [12]. In contrast, our study, which included all nodules regardless of their TIRADS classification, demonstrated superior diagnostic performance. In addition, head-to-head evidence from included studies consistently showed AI matching or surpassing human readers in AUC, sensitivity, and specificity, highlighting its ability to reduce inter-observer variability and enhance diagnostic consistency across centers. The higher accuracy observed in our analysis likely reflects the ability of machine learning algorithms to capture complex textural and morphological features beyond human visual perception, thus improving reproducibility and reducing interobserver variability. Importantly, this approach is not subject to the selection bias that may arise when lesions are categorized solely on the basis of visual ultrasound assessment.

When comparing machine learning performance according to the reference standard adopted (cytological versus histological findings), no statistically significant differences were demonstrated. Although slightly higher pooled estimates were observed in studies using cytology, the mixed-effects meta-regression analysis did not confirm a significant association with diagnostic accuracy (p = 0.238 for sensitivity; p = 0.188 for specificity). This result suggests that the choice of reference standard did not substantially influence the overall diagnostic performance of the evaluated models, supporting the robustness of machine learning algorithms across different validation settings.

From a clinical perspective, AI could improve the selection of nodules requiring FNA, thereby reducing unnecessary procedures, associated complications, and healthcare costs. Although FNAC is generally considered simple, reliable, safe, and well-accepted by patients, it is not entirely free from complications [56]. Minimizing its overuse remains an important goal in precision thyroid care.

Despite these promising results, several methodological and clinical issues limit large-scale implementation. Most existing algorithms are trained on single-center or ethnically homogeneous datasets, potentially reducing their applicability to broader populations and imaging devices. Furthermore, the intrinsic “black-box” nature of deep learning models continues to hinder interpretability and clinical trust, as the relative contribution of sonographic features—such as microcalcifications, vascularity, or stiffness—remains largely opaque.

Nevertheless, AI offers clear advantages over conventional risk stratification systems, including improved standardization, quantitative feature extraction, and enhanced diagnostic consistency. When adequately validated, these tools could assist radiologists in achieving more objective and reproducible image interpretation. Future research should aim to integrate AI into multimodal clinical workflows, combining ultrasound, cytological, and molecular data, while ensuring transparency, explainability, and equity across diverse patient populations.

Finally, some limitations of the present study should be acknowledged. First, all included studies were conducted in Asia. Although based on a large patient population, the findings may not be representative of the general population due to differences in genetic background, dietary habits, and iodine intake. Second, a substantial proportion of studies relied on public datasets for external validation. These open-source datasets often lack comprehensive clinical and, in particular, imaging information, potentially reducing the robustness and reliability of the results. In addition, most included studies did not report whether follow-up imaging was used as an adjunct reference standard for low-risk nodules (TIRADS 1–3). Consequently, the datasets likely over-represent nodules that underwent cytology or post-surgical histopathological evaluation (typically TIRADS 4–5), while under-representing nodules managed through surveillance. This limitation may reduce the representativeness of the included cohorts and should be taken into account when interpreting diagnostic accuracy. Finally, the underrepresentation of pathological subtypes—with most studies focusing on classical papillary thyroid cancer—has led to diagnostic inequity for patients with follicular thyroid cancer and other rare variants. This limitation may impair the generalizability of algorithmic performance across institutions, imaging devices, and multiethnic cohorts. We must also underline that an additional major limitation of our meta-analysis is the inconsistent reporting of key methodological variables across the included studies. In particular, information on TIRADS score distribution, the use of machine learning versus deep learning approaches, the specific deep learning architectures adopted, whether nodule segmentation was performed manually or automatically, and the histologic subtype was rarely provided in a standardized manner. The absence of these data prevented more detailed subgroup analyses and meta-regressions that might have explained part of the heterogeneity observed in the pooled accuracy estimates

Finally, the ‘black-box’ nature of AI models remains a major obstacle to clinical implementation. Current systems still fall short of providing transparent insights into their decision-making process. In particular, they fail to adequately explain the relative importance of key morphological features, such as microcalcifications versus vascular patterns [57], [58], [59], [60], [61]. This lack of interpretability hampers the clinical validation of AI outputs, especially in cases of misclassification—for example, the erroneous identification of Hashimoto’s thyroiditis as thyroid malignancy.

In conclusion, this meta-analysis confirms that AI-based ultrasound models achieve high diagnostic accuracy in differentiating benign from malignant thyroid nodules, with performance metrics consistently validated across independent datasets. Their consistent performance across validation frameworks supports their potential integration into routine clinical workflows, paving the way for a more objective, reproducible, and data-driven approach to thyroid nodule management.

## CRediT authorship contribution statement

**Claudio Casella:** Visualization, Validation, Supervision. **Carlo Cappelli:** Visualization, Validation, Supervision, Conceptualization. **Roberto Gatta:** Writing – original draft, Data curation. **Elisa Gatta:** Writing – original draft, Formal analysis, Data curation. **Samuele Isoli:** Writing – original draft, Data curation. **Riccardo Morandi:** Writing – review & editing. **Simone Vetrugno:** Writing – review & editing. **Sara Corvaglia:** Writing – review & editing. **Ilenia Pirola:** Visualization, Validation, Supervision. **Virginia Maltese:** Writing – review & editing.

## Ethical statement

As this work is a systematic review and meta-analysis based on previously published studies, it did not involve direct patient recruitment or the use of individual patient data. Therefore, approval from an institutional ethics committee was not required.

## Declaration of Competing Interest

The authors declare that they have no known competing financial interests or personal relationships that could have appeared to influence the work reported in this paper.
